# Targeting peripheral opioid receptors to promote analgesic and anti-inflammatory actions

**DOI:** 10.3389/fphar.2013.00132

**Published:** 2013-10-24

**Authors:** Katerina S. Iwaszkiewicz, Jennifer J. Schneider, Susan Hua

**Affiliations:** The School of Biomedical Sciences and Pharmacy, The University of NewcastleCallaghan, NSW, Australia

**Keywords:** pain, inflammation, peripheral opioid receptors, opioids, immune cells, analgesia, anti-inflammatory

## Abstract

Mechanisms of endogenous pain control are significant. Increasing studies have clearly produced evidence for the clinical usefulness of opioids in peripheral analgesia. The immune system uses mechanisms of cell migration not only to fight pathogens but also to control pain and inflammation within injured tissue. It has been demonstrated that peripheral inflammatory pain can be effectively controlled by an interaction of immune cell-derived opioid peptides with opioid receptors on peripheral sensory nerve terminals. Experimental and clinical studies have clearly shown that activation of peripheral opioid receptors with exogenous opioid agonists and endogenous opioid peptides are able to produce significant analgesic and anti-inflammatory effects, without central opioid mediated side effects (e.g., respiratory depression, sedation, tolerance, dependence). This article will focus on the role of opioids in peripheral inflammatory conditions and the clinical implications of targeting peripheral opioid receptors.****

## INTRODUCTION

Inflammation is the series of highly coordinated events that is the response of vascular tissues to detrimental stimuli which results in heat, swelling, redness, and pain (Machelska et al., 2003). It is a vital protective response that functions to provide a rapid response to injuries to stimulate repair and prevent further disturbance to the affected tissue (Stein et al., 2003). Acute peripheral inflammatory pain is associated with immediate immune cell infiltration following tissue damage. Several disorders are correlated with peripheral inflammatory pain, such as arthritis and soft tissue injuries. Hyperalgesia, which is a lowered threshold to pain, is also associated with inflammatory pain. After tissue damage, resident macrophages generate an inflammatory response via release of mediators. These cause local inflammatory changes as well as attract more leukocytes to the site of inflammation which amplify these changes. Inflammatory mediators that are released and tissue acidification activate nociceptive primary afferent neurons as well as lower their signaling threshold in order to stimulate the sensation of pain and cause hyperalgesia (Hua and Cabot, 2010).

## ROLE OF ENDOGENOUS OPIOID SYSTEMS IN INFLAMMATION

An excess of pain or long-lasting pain (not necessarily “chronic”) is counterproductive and so the body utilizes endogenous analgesic compounds to alleviate this sensation (Stein, 2013). Endogenous pain control mechanisms are not limited to the central nervous system (CNS). Although central mechanisms play a prominent role, evidence in the literature also suggests a significant involvement of peripheral mechanisms in counteracting pain. In fact, peripheral analgesic mechanisms have been demonstrated both in animals and humans, especially under inflammatory conditions. Most of these involve the release of opioid peptides, endocannabinoids, somatostatin, or anti-inflammatory cytokines (Przewlocki et al., 1992; Luster et al., 2005; Hua and Cabot, 2010). Opioid peptides and their roles in anti-nociception have been extensively examined and their clinical relevance has been demonstrated in both human and animal studies (Busch-Dienstfertig and Stein, 2010; Stein, 2013).

Opioid-mediated analgesia is instigated in both the CNS and periphery through the release of endogenous opioid peptides. Evidence from the literature has shown that the central and peripheral mechanisms of endogenous opioid analgesia are interconnected, particularly in the early stages of inflammation (Binder et al., 2004; Brack et al., 2004d; Labuz et al., 2007; Hua and Cabot, 2010). The later stages have shown to have an increased role for peripheral opioid antinociception (Labuz et al., 2006, 2007). In the periphery, immune cells have been demonstrated to contain and release the opioid peptide β-endorphin in inflamed tissues which then acts upon opioid receptors present on primary afferent neurons to block pain transmission and thus provide analgesia (Mousa et al., 2000; Vetter et al., 2006). Labuz et al. (2006) demonstrated that treatment of inflamed tissue with anti-β-endorphin antibody attenuated endogenous antinociceptive effects in this tissue. This local opioid-mediated effect is limited to the periphery and thus does not have the adverse systemic effects of centrally mediated opioid analgesia (Labuz et al., 2006).****

## OPIOID PEPTIDES AND RECEPTORS IN INFLAMMATION

There are three families of opioid peptides that have been extensively studied: the endorphins, enkephalins and dynorphins, each of which is derived from a distinct gene and the precursors, pro-opiomelanocortin (POMC), pro-enkephalin (PENK) and prodynorphin (Hua and Cabot, 2010; Busch-Dienstfertig and Stein, 2010). Once released from immune cells opioid peptides act upon opioid receptors that are located on primary afferent neurons and have been shown to be co-localized with nociceptors (Joseph and Levine, 2010; Wang et al., 2010; Stein, 2013). Opioid peptides render nociceptors less sensitive to excitation and thus impede the action of multiple excitatory mediators in one step. Each opioid peptide does not bind exclusively to one unique opioid receptor but instead exhibits affinity for various opioid receptors including μ-, ∂- and κ-opioid receptors. During states of inflammation the numbers of opioid receptors in the periphery have been noted to be increased. For example, Jeanjean et al. (1995) showed that a single intraplantar injection of interleukin-1β (IL-1β) was able to enhance the axonal transport of mu and kappa opioid receptors in the injected paw for as long as 6 days following a single injection. This increase in receptors is due to an increase in receptor synthesis, not an increase in speed of axonal transport, and it is theorized to occur through IL-1β stimulated retrograde axonal transport of cytokines and nerve growth factor from the inflamed tissue that alter neuronal gene expression in dorsal root ganglia neurons (Jeanjean et al., 1995; Puehler et al., 2004; Busch-Dienstfertig and Stein, 2010).

Enzymes within neurons such as phosphokinase C can phosphorylate opioid receptors, which leads to an increased affinity for arrestin molecules. Opioid-arrestin complexes have a decreased sensitivity for extracellular opioid peptides and are likely to be internalized via clathrin-dependent pathways (Law et al., 2000; Busch-Dienstfertig and Stein, 2010). From here, the receptors can either be recycled by reinsertion into the membrane, or they can be degraded. Recycling of peripheral opioid receptors has been shown to avoid the development of tolerance to opioids in the periphery via prevention of desensitization (Koch et al., 2005). Opioid peptides also work to prevent vesicular release from neurons via inhibition of ion channels, which prevents the release of excitatory neuropeptides such as noradrenaline and substance P (Stein, 2013). As tolerance is a significant problem associated with systemic administration of opioids, the targeting of peripheral opioid receptors, which have innate mechanisms to prevent desensitization, is clinically relevant (Joseph et al., 2010).

## OPIOID-CONTAINING IMMUNE CELLS IN INFLAMMATION

Inflammation rapidly stimulates immune cell extravasation and migration into tissues (Machelska et al., 2004; Rittner and Stein, 2005). Quantitative analysis has revealed that in early inflammation granulocytes (esp. neutrophils) are the major opioid-containing leukocyte, whereas at later stages of inflammation, monocytes/macrophages and lymphocytes (esp. activated T- and B-cells) predominate (Rittner et al., 2001; Brack et al., 2004a,b; Machelska et al., 2004). This is consistent with their order of infiltration to inflamed tissues (Luster et al., 2005). Inflammation has been shown to increase the expression of opioid peptides within these cells. In fact all opioid peptides as well as their mRNA transcripts encoding their precursor proteins have been identified within immune cell, with β-endorphin from POMC being the most prominent (Cabot et al., 1997; Cabot, 2001; Mousa et al., 2001). However, it has been suggested that only a finite number of the total immune cell population actually produce opioid peptides and home to lymph nodes (Stein et al., 2003; Rittner et al., 2005). This is supported by the observation that β-endorphin and POMC mRNA were less abundant in circulating lymphocytes than in those in lymph nodes (Cabot et al., 1997). Similarly, Przewlocki et al. (1992) demonstrated the presence of immune cells containing the opioid peptide precursors, POMC and PENK, in inflamed tissue and the absence of these mRNA in non-inflamed tissue. Labuz et al. (2006) identified that the majority of immune cells infiltrating inflamed tissue contained β-endorphin. Therefore with the duration of inflammation, the number of infiltrating immune cells as well as total opioid peptide content at the site of tissue injury increases. These opioid peptides are translated and processed at the site of inflammation within immune cells.

## MIGRATION OF OPIOID-CONTAINING LEUKOCYTES TO INFLAMED TISSUE

**FIGURE 1:**
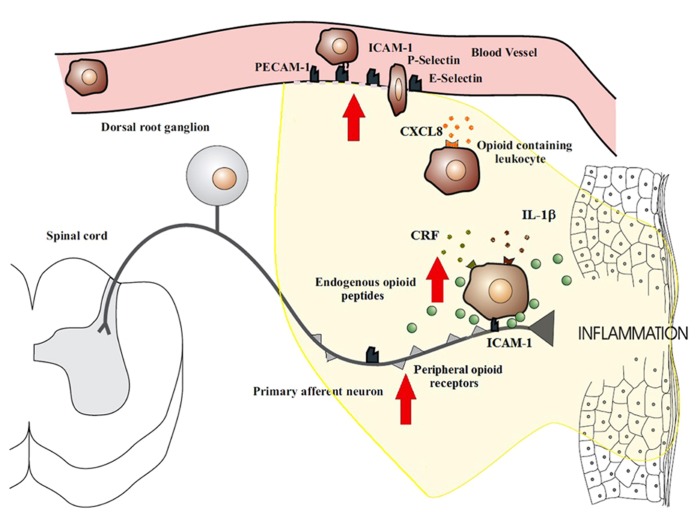
**Migration of opioid-containing immune cells and opioid release within inflamed tissue.** Adhesion molecules interact with their respective ligands to facilitate endothelial transmigration of immune cells. In response to stress or releasing agents (e.g., CRF, IL-1, CXCL8), the immune cells secrete opioid peptides. Opioid peptides or exogenous opioids bind to opioid receptors on primary afferent neurons, leading to analgesia. Direct adhesion between opioid-containing immune cells and peripheral sensory neurons (via ICAM-1 and/or NCAM) may be necessary to release opioid peptides within the effective range of peripheral opioid receptors. The immune cells, depleted of opioids, then migrate to regional lymph nodes. The arrows denote an increased expression within inflamed tissue of cell adhesion molecules, opioid receptors, endogenous opioid peptides, and receptors for ligands that trigger opioid release on the surface of immune cells (e.g., CXCR2, IL-1 receptors, CRF receptors). This all enhances the analgesic and anti-inflammatory activity of the peripheral opioid pathway in inflammatory conditions.

Peripheral tissue injury causes a migration of opioid peptide-containing immune cells to the inflamed site. This migration appears to be both centrally and locally regulated. Exogenously stimulated systemic inhibition of pain has been shown to decrease the recruitment of β-endorphin containing immune cells in inflamed tissue (Schmitt et al., 2003; Heurich et al., 2007), thus suggesting a role for central regulation. Local regulation has been more extensively studied and involves a series of binding events to endothelial cells. The trafficking of opioid-containing immune cells occurs in a site-directed manner since they express adhesion molecules that govern their recruitment to damaged tissue (Schmitt et al., 2003). The homing of leukocytes to inflamed tissues involves a precise series of events which begin with circulating leukocytes tethering to and rolling along the vascular endothelial cell wall in a process mediated by P-, E-, and L-selectins (Koning et al., 2002; Stein et al., 2003; **Figure 1**). Upregulation of these cell adhesion molecules has been noted in many models of inflammation (Schurmann et al., 1995; Krishna et al., 1997; Navarro et al., 2002; Almulki et al., 2009; Svensson et al., 2009). Leukocytes are then activated by chemokines released from inflammatory cells and presented on the luminal surface of the endothelium (Koning et al., 2002; Machelska et al., 2002; Stein et al., 2003). This causes an increase in the avidity as well as an up-regulation of leukocyte integrins, in particular CD49d/CD29 and CD18, which mediate the firm adhesion of leukocytes to endothelial cells by interacting with intercellular adhesion molecule-1 (ICAM-1; Koning et al., 2002; Machelska et al., 2002; Stein et al., 2003). Leukocytes then migrate through the endothelium with the aid of platelet-endothelial cell adhesion molecule-1 (PECAM-1) expressed on endothelial cells at intercellular junctions. All these molecules are constitutively expressed and are upregulated in inflammation, except L-selectin, which is rapidly shed upon activation (Koning et al., 2002; Muro and Muzykantov, 2005). The up-regulation of these necessary components of leukocyte transmigration in inflammation results in the increased influx of immune cells. The preferential increase in opioid-containing immune cells can be explained by the co-expression of L-selectin, integrin-β and the chemokine receptor CXCR2 on opioid-containing leukocytes (Mousa et al., 2000; Brack et al., 2004a,b,c,d; Machelska et al., 2004). Pre-treatment of rats with antibodies against these molecules have been shown to significantly decrease the number of opioid-containing immune cells that accumulate in inflamed tissue (Machelska et al., 1998, 2002, 2004).

## RELEASE OF OPIOID PEPTIDES FROM LEUKOCYTES

Opioid-containing immune cells release opioid peptides within peripheral inflamed tissue before moving to the local lymph node (Hua and Cabot, 2010). The release of opioids has been demonstrated to be governed by various factors. The chemokine CXCL8 (also known as IL-8) in humans which acts upon CXCR2 has likewise been demonstrated to increase release of opioid peptides *in vitro*. CXCL8-mediated opioid release occurs via binding of CXCL8 to its receptor on the leukocyte, which causes vesicular release of opioid peptides in a calcium-dependant manner (Rittner et al., 2006). This is the same process that is used by other inflammatory factors such as corticotropin-releasing factor (CRF) and IL-1β.

Schafer et al. (1997) demonstrated the ability of CRF to cause opioid release *in vitro* from immune cells obtained from inflamed tissue. Labuz et al. (2006) through the use of anti-β-endorphin antibody *in vivo* showed that release of β-endorphin from immune cells is mediated by CRF interacting with its respective receptor on leukocytes. CRF and its respective receptors on the surface of leukocytes are upregulated in inflammation (Schafer et al., 1997). Schafer et al. (1997) further demonstrated that IL-1β influences the release of β-endorphin from immune cells both *in vitro* and *in vivo* when administered locally via receptor binding. IL-1β levels increase during inflammation and enacts a positive feedback loop in order to increase its own concentration, whilst also having pro-inflammatory effects by upregulating the release of inflammatory factors (Schafer, 2003; Jimbo et al., 2005). Therefore as inflammation progresses, the level of endogenous opioid release also increases.

β-endorphin containing inflammatory cells situated in close proximity to sympathetic nerve fibres within inflamed tissue have also been observed to possess increased numbers of α1- and β2-adrenergic receptors. Destruction of these receptors was demonstrated to abolish endogenous opioid analgesia, thus suggesting that noradrenaline release by neuronal cells may also stimulate the release of opioid peptides (Binder et al., 2004; Machelska et al., 2004).

## THE EFFECT OF INFLAMMATORY CONDITIONS ON OPIOID EFFICACY

The rate of metabolic degradation of opioid peptides is increased in inflamed tissues (Vetter et al., 2006; Schreiter et al., 2012). Release of inflammatory factors such as hydrogen ions from damaged cells, cytokines and chemokines from resident cells and peptidases from immune and neuronal cells create a hostile environment that acts to quickly break down opioids (Vetter et al., 2006; Schreiter et al., 2012). In order for opioid peptides to reach their receptors on nociceptive primary afferent neurons, interaction between neurons and immune cells may be required.

Recent reports have described bidirectional communication between immune and neuronal cells as well as physical contact between these cells. Of note is the observation of peripheral nerves and opioid-containing immune cells being closely associated (Przewlocki et al., 1992; Hua et al., 2006; Hua and Cabot, 2010). *In vitro* studies have demonstrated consistent alliance between lymphocytes containing opioids and cultured DRG nerves (Hua et al., 2006) whilst *in vivo* studies has observed this same phenomena in peripheral inflamed tissues with primary afferent nerves (Przewlocki et al., 1992). The mechanics of this association are yet to be elucidated, but is likely to be either by a synaptic-like connection being formed or paracrine opioid release. Adhesion molecules come into play with both of these processes in order to form intercellular interactions that are stable and cell specific (Dustin and Colman, 2002; Yamagata et al., 2003). Inhibition of ICAM-1 and neural cell adhesion molecules (NCAM) have both been demonstrated to result in a decrease lymphocyte–DRG neuronal cell interactions (Hua et al., 2006), which suggests that these play an important role in immune and neuronal cell adhesion. Whilst the exact mechanisms of these interactions are not yet understood, they may play a necessary role in the development of effective endogenous analgesia by ensuring delivery of opioids to peripheral sensory neurons before they are degraded by the extracellular environment within inflamed tissues (**Figure 1**).

## ANTI-INFLAMMATORY EFFECT OF PERIPHERAL OPIOIDS

Endogenous opioids may enact anti-inflammatory effects as well as analgesia through their actions on neuronal cells through prevention of vesicular release of noradrenaline and substance P. The function of noradrenaline in inflammation is contested, with evidence being provided for both a positive and negative role. Schlachetzki et al. (2010) observed enhanced expression of COX-2, which is heavily implicated in inflammation, following treatment with noradrenaline. However, data has also been presented that correlates noradrenaline release in Alzheimer’s disease with suppression of neuroinflammation (Heneka et al., 2010). Substance P conversely has a well-reviewed pro-inflammatory action (O’Connor et al., 2004). Walker (2003) suggests that an opioid-mediated reduction of tumour necrosis factor (TNF) production and release also contributes to anti-inflammatory actions. TNF is a known regulator of inflammation and its inhibition has been shown to be an effective anti-inflammatory treatment (Esposito and Cuzzocrea, 2009). As the process of blocking the release of these neuropeptides is due to opioid receptor mediated hyperpolarisation of neuronal cells, exogenous opioids theoretically should have the same anti-inflammatory effect.

An opioid receptor independent mechanism may also be involved in opioid-mediated anti-inflammation. Gavalas et al. (1994) showed that experimentally induced mouse paw oedema was significantly inhibited after the administration of opioids and this effect was not reversed by naxolone. Conversely, Philippe et al. (2003) noted that naxolone was able to reverse the mu-opioid receptor mediated reduction in inflammation in two *in vivo* models of colitis. These results suggest that a variety of complex regulatory activities may be performed by opioid agonists in various tissues of the body that may be naloxone-sensitive or naloxone insensitive and these pathways may directly or indirectly inhibit the release of cytokines and mediators involved in inflammation (Gavalas et al., 1994).

Fecho et al. (2007) demonstrated an anti-inflammatory action of morphine through the reduction of swelling and accumulation of neutrophils in carrageenan-induced peripheral inflammation. This effect was not dose-dependent and was not reversed by naloxone. The anti-inflammatory effect displayed by morphine is likely due to modulation of the adherence of immune cells to the endothelium by affecting the expression of cell adhesion molecules, and consequently affecting leukocyte transmigration. In comparison, administration of liposomes loaded with loperamide HCl and conjugated with antibody to intercellular adhesion molecule-1 (anti-ICAM-1), exerted analgesic and anti-inflammatory effects exclusively in peripheral painful inflamed tissue when administered intravenously in the complete Freund’s adjuvant (CFA) model of acute inflammatory pain (Hua and Cabot, 2013). It was demonstrated previously that this adhesion between opioid-containing lymphocytes and cultured sensory neurons was opioid receptor dependent, with naloxone inhibiting the reduced neuroimmune adhesion in the presence of beta-endorphin (Hua et al., 2006). Opioids have been found to have significant anti-inflammatory effects in peripheral inflamed tissues, thus they are of clinical significance in relation to the treatment of peripheral inflammatory pain.

## CLINICAL IMPLICATIONS AND PERSPECTIVES

### TOPICAL OPIOID DELIVERY

Topically applied opioids have already been employed as a way of instigating peripheral nociception, however further studies are required to ascertain the anti-inflammatory activity of topical opioids. Case studies have reported the effectiveness of morphine when combined with pre-made gels such as IntraSite^®^ for use in ulcers. van Ingen et al. (2010) recorded a case where topical morphine gel was applied to a cutaneous ulcer that had previously been treated unsuccessfully with zinc oil and surgical debridement. Within 3 days of the start of morphine gel treatment, the visual analog scale (VAS; 0–100) for pain, as determined by the patient, was halved from 80 to 40 (van Ingen et al., 2010). Welling (2007) explored the use of topical morphine in burns patients as a way of providing analgesia and avoiding systemic side effects, however no significant evidence of analgesia was seen when compared to the control groups. This study had a low level of power with a small number of participants, and damaged neuronal cells in burn patients may also account for the lack of efficacy in comparison to studies on chronic inflammatory wounds. This may be due to damaged nerve endings that result in impaired signal transduction or receptor expression. Loperamide has been reported upon in relation to the treatment of thermal injury in animals. Topical delivery of loperamide was used by Nozaki-Taguchi and Yaksh (1999) to successfully initiate antihyperalgesia in rats after thermal injury (Nozaki-Taguchi and Yaksh, 1999). In humans, loperamide has been used in solution for analgesia in graft-versus-host related oral pain (Nozaki-Taguchi et al., 2008). Nozaki-Taguchi et al. (2008) studied four patients who were suffering from oral pain following blood stem cell transplantation. Utilizing the loperamide mouthwash resulted in oral analgesia and reduction of hyperalgesia in these patients. Analgesia was not noted in any other parts of the body, but some inhibition of gut motility was exhibited (Nozaki-Taguchi et al., 2008).

### PARENTERAL OPIOID DELIVERY

Peripheral mechanisms of opioid analgesia are well-established in the literature and have gained recognition in the clinical setting**in conditions such as chronic rheumatoid arthritis and osteoarthritis, bone pain, and postoperative pain after laparoscopic, urinary bladder, and knee surgery (Busch-Dienstfertig and Stein, 2010). In patients undergoing arthroscopic knee surgery, opioid-containing immune cells are detectable in the inflamed synovium and the blockade of intra-articular opioid receptors by naloxone results in significantly increased postoperative pain for up to 4 h (Stein et al., 1993). More recently, peripheral opioid analgesia has also been reported in experimental neuropathic pain (Labuz et al., 2009, 2010).

The most extensively studied clinical situation is the intra-articular application of opioid agonists for pain control after knee surgery (Stein and Lang, 2009; Busch-Dienstfertig and Stein, 2010), which is now established in routine clinical practice (Lang et al., 2010). Intra-articular morphine was found to be as potent as dexamethasone in reducing pain as well as synovial inflammation (Stein et al., 1999). However, a limitation of intra-articular administration of opioids is that repeated injections carry a risk of infection and cannot be easily applied to more than one joint. Negative results from some reviews have been attributed to lack of study sensitivity, lack of tissue inflammation, or the superimposition of general or local anesthetic effects (Stein and Machelska, 2011). Importantly, the use of peripherally acting opioid agonists for the prolonged treatment of inflammatory pain has not been shown to induce peripheral tolerance (Zollner et al., 2008; Lang et al., 2010), which has important implications for the treatment of pain associated with conditions such as chronic arthritis, inflammatory neuropathy, postoperative pain and cancer. Unfortunately the clinical studies to date were not designed to focus on the anti-inflammatory potential of opioids, as this is a more recent established finding of peripheral opioid therapy. Therefore, future clinical studies should incorporate both quantitative and qualitative anti-inflammatory measures into the research design.

## CONCLUSION

Increasing studies have clearly produced evidence for the clinical usefulness of peripheral opioid analgesics, in particular administration of opioids into local inflamed tissue. A major long-term goal remains to develop peripherally selective opioid compounds, suitable for oral and/or intravenous route of administration to improve clinical pain relief. Peripherally targeted opioids may be used in a co-treatment approach with other therapies (e.g., cytokine modulators or TNF-α antagonists) at low doses for conditions such as rheumatoid arthritis. This may provide a much sought after alternative for the management of chronic arthritic and other inflammatory conditions whilst avoiding central opioid mediated side effects and of typical side effects produced by non-steroidal and steroidal anti-inflammatory drugs.

## Conflict of Interest Statement

The authors declare that the research was conducted in the absence of any commercial or financial relationships that could be construed as a potential conflict of interest.
